# Barriers to connecting with the voluntary assisted dying system in Victoria, Australia: A qualitative mixed method study

**DOI:** 10.1111/hex.13867

**Published:** 2023-09-11

**Authors:** Ben P. White, Ruthie Jeanneret, Lindy Willmott

**Affiliations:** ^1^ Australian Centre for Health Law Research, Faculty of Business and Law Queensland University of Technology Brisbane Queensland Australia

**Keywords:** access to health care, assisted dying, euthanasia, medical assistance in dying, patient experience, qualitative study

## Abstract

**Introduction:**

Voluntary assisted dying (VAD) is increasingly being legalised internationally. In Australia, all six states have now passed such laws, with Victoria being the first in 2019. However, early research in Victoria on the patient experience of seeking VAD shows that finding a connection to the VAD system is challenging. This study analyses the causes of this ‘point of access’ barrier.

**Methods:**

We conducted semi‐structured qualitative interviews with family caregivers and a person seeking VAD, with participants recruited via social media and patient interest groups. Data were thematically analysed. We also undertook documentary analysis (content and thematic) of publicly available reports from the oversight body, the Voluntary Assisted Dying Review Board.

**Results:**

We interviewed 32 family caregivers and one patient across 28 interviews and analysed six Board reports. Finding a point of access to the VAD system was reported as challenging in both interviews and reports. Four specific barriers to connecting with the system were identified: (1) not knowing VAD exists as a legal option; (2) not recognising a person is potentially eligible for VAD; (3) not knowing next steps or not being able to achieve them in practice; and (4) challenges with patients being required to raise the topic of VAD because doctors are legally prohibited from doing so.

**Conclusion:**

Legal, policy and practice changes are needed to facilitate patients being able to find a connection to the VAD system. The legal prohibition on doctors raising the topic of VAD should be repealed, and doctors and institutions who do not wish to be involved in VAD should be required to connect patients with appropriate contacts within the system. Community awareness initiatives are needed to enhance awareness of VAD, especially given it is relatively new in Victoria.

**Patient or Public Contribution:**

Families and a patient were the focus of this research and interviews with them about the experience of seeking VAD were the primary source of data analysed. This article includes their solutions to address the identified point of access barriers. Patient interest groups also supported the recruitment of participants.

## 1 INTRODUCTION

There is an international trend to legalise voluntary assisted dying (‘VAD’), also known as euthanasia, physician‐assisted dying, or medical assistance in dying.^1^ Australia has also witnessed rapid reform. Over the last 6 years, all six Australian states have separately legalised VAD, with the two territories set to follow.^2^

There is a distinctive Australian model of VAD.^3^ It is a conservative model with many safeguards, most of which are prescribed in detailed and lengthy legislation, and narrow eligibility criteria, confining access to terminally ill adults expected to die within a limited timeframe. This study focuses on the VAD system in the Australian state of Victoria (the first state to permit VAD). The Voluntary Assisted Dying Act 2017 (Vic) (‘Victorian law’) commenced in June 2019 after an 18‐month implementation period.^4^

VAD systems, including Victoria's, aim to provide choices for eligible patients who want assistance to die. A second aim, which can be in tension with the first, is ensuring VAD systems operate safely by excluding ineligible people from accessing VAD and protecting the community, particularly the vulnerable.^5^ While much focus has been on system safety, there is increasing scrutiny on the Victorian system's accessibility.^5^, ^6^, ^7^, ^8^, ^9^, ^10^, ^11^, ^12^ A recent study of Victorian family caregivers' perceptions of their family member's experience seeking VAD identified a series of access barriers: finding doctors to assess eligibility; the time the approval process took; institutional objection; and a legal prohibition on health practitioners raising VAD with patients.^6^

A key finding from that study was that once patients and families were connected with the Victorian VAD system, either through a willing doctor or VAD Care Navigator (see below), the process flowed well. However, until then, patients and families felt lost and unclear about how to seek VAD. This is consistent with international experience. For example, Canadian studies report frustration and confusion by patients, families, and healthcare providers about pathways to access assisted dying.^13^, ^14^, ^15^, ^16^

This paper looks beyond existing research into general barriers to accessing VAD and focuses on *specific* barriers to the ‘point of access’, namely when a patient transitions from outside the VAD system to being connected with it. Reflecting wider literature about health literacy barriers to healthcare,^17^ this access point is critical because an inability to connect to the VAD system precludes eligible patients from making this now lawful choice. To our knowledge, this paper is the first internationally to specifically investigate this point of access to VAD, although we acknowledge resonance with Crumley et al.'s^14^ ‘starting the process’ step in the Canadian assisted dying process.

To understand the Victorian VAD process, we outline the Victorian law and system in Box 1. However, we specifically mention four key aspects for understanding ‘point of access’ to VAD. The first is that registered health practitioners are prohibited by law from raising VAD with patients.^19^, ^20^ This means VAD must be patient‐initiated. Second, the law protects registered health practitioners' rights to conscientiously object to being involved in VAD,^19^ but does not require practitioners to refer to another practitioner or provide information about VAD. Third, the law permits only doctors who have particular qualifications and have undertaken the state‐approved VAD training to be involved in VAD.^19^ At least one assessing doctor must have expertise and experience in the patient's disease.^19^

BOX 1 **Overview of Victorian VAD Act^a^**

**Table:** 

Aspect of process	Summary of VAD Act provisions
Discussions about VAD (*Section 8 of the VAD Act*)	Registered health practitioners cannot initiate discussions about VAD or suggest VAD to a person in the course of providing health or personal care services.
Eligibility criteria (*Section 9 of the VAD Act*)	To be eligible for access to VAD, a person must: 1. be aged 18 years or more; 2. be an Australian citizen or permanent resident; 3. be ordinarily resident in Victoria for at least 12 months at the time of making a first request; 4. have decision‐making capacity in relation to VAD; 5. be diagnosed with a disease, illness or medical condition that is: − incurable; − advanced, progressive and will cause death; − is expected to cause death within 6 months (or 12 months, for neurodegenerative diseases, illnesses or medical conditions); and − is causing suffering to the person that cannot be relieved in a manner the person considers tolerable.
Request and assessment process (*Part 3 of the VAD Act*)	A person may access VAD if: 1. they have made three requests for VAD (a first request, written declaration, and final request); 2. they have been assessed as eligible for access to VAD by two eligible medical practitioners who have completed mandatory training (called the co‐ordinating medical practitioner, who completes the first assessment, and the consulting medical practitioner, who completes the consulting assessment); 3. they have appointed a contact person; 4. the co‐ordinating medical practitioner has completed the final review, certifying the process was in accordance with the VAD Act; and 5. they have been issued with a permit by the Secretary of the Department of Health (see below). The person must also understand specific information (e.g., about prognosis, treatment and palliative care options), and their request must be enduring, and they must be acting voluntarily and without coercion. The written declaration must be witnessed by two independent, eligible witnesses. The person must have decision‐making capacity, including at the time of administration.
Permit approval (*Part 4 of the VAD Act*)	A self‐administration or practitioner administration permit must have been issued in relation to the person by the Secretary of the Department of Health before the person is authorised to access VAD.
Administration (*Part 5 of the VAD Act*)	The default method of administration is self‐administration. Practitioner administration is only permissible if the person is physically incapable of self‐administering or digesting the VAD substance. For practitioner administration, the person must make an administration request and administration must be witnessed by an independent witness. The VAD substance is managed by a coordinated, statewide pharmacy service. The contact person has obligations in relation to the VAD substance, including returning any unused or remaining VAD substance to the dispensing pharmacy.
Oversight (*Part 9 of the VAD Act*)	The Act establishes the VAD Review Board as the oversight body to review VAD cases in Victoria. Forms must be completed and uploaded at to an electronic portal at certain steps in the process and are reviewed by the VAD Review Board. Some decisions made during the request and assessment process are reviewable by the Victorian Civil and Administrative Tribunal. The contact person is contacted by the VAD Review Board to provide feedback and has other reporting obligations.

Abbreviations: VAD, voluntary assisted dying.

^a^ Table is adapted from Jeanneret et al.^18^

A final noteworthy feature of the VAD system is the Statewide VAD Care Navigator Service (‘Care Navigators’): state‐funded health practitioners who support people seeking VAD.^21^ The Care Navigators expanded from two navigators in Melbourne (Victoria's capital) to now include Victoria's five rural health regions. Some hospitals and health services also appointed local navigators or coordinators to support their patients seeking VAD. These navigators are sometimes the first contact point in the system in Victoria (and internationally^14^, ^16^), hence are important for locating a ‘point of access’. While navigators do not assess eligibility criteria, they help locate willing and trained doctors to assess patients, including through their nonpublic register of participating doctors.

## 2 METHODS

This study draws on two sources. The first is semi‐structured qualitative interviews about patients' experience of seeking VAD, as reported (primarily) by family caregivers. The second is an analysis of publicly available reports from the Victorian oversight body, the Voluntary Assisted Dying Review Board (‘Board’). We used this methodological triangulation to test findings and obtain a more complete picture of point of access issues in Victoria's VAD system.^22^

### 2.1 Semi‐structured interviews

#### 2.1.1 Sampling and recruitment

Eligible participants were (a) patients seeking VAD in Victoria (whether or not they had been approved for VAD), and (b) family caregivers who had or were supporting patients through this process (hence can directly report on their perceptions of patient experience). Participants were recruited through research team Twitter posts and patient interest groups Go Gentle Australia and Dying with Dignity Victoria, who shared study details via social media, newsletters, and emails. Initially relying on convenience sampling, we later used purposive sampling specifying missing perspectives such as those who sought VAD but were not approved, including via Twitter and direct emails from the above groups to potentially matching participants. We sought a breadth of domains (from the relevant Board report)^23^ including patient age, gender, illness, location (metropolitan/regional), timing of seeking access, and patient experience of VAD (self‐administration, practitioner administration, sought VAD but did not use or not approved).

#### 2.1.2 Data collection

Our interview guide (Supporting Information File) was based on analysis of Victorian law,^5^ previous interviews with doctors providing VAD,^9^, ^10^, ^12^ and iterative research team discussion. The guide reflected the VAD process chronologically and family caregivers were asked to focus on the patient's experience. ‘Point of access’ to the VAD system was specifically explored including these questions:

1. When did you first become aware of VAD and that it might be possible?
2. Was it easy to get the information you wanted about VAD or were there barriers?
3. When did you first talk about VAD with a doctor or health professional?
4. Did you know that health professionals can't raise VAD with you first?

Participants provided free and informed consent. Interviews were conducted by B. P. W. (health law and regulation professor) and R. J. (lawyer and PhD student) together, with one a designated lead. Interviews occurred between 17 August and 26 November 2021 via Zoom videoconferencing except for two by phone and one in‐person. Recruitment ceased on data saturation (information redundancy).^24^ Interviews were digitally audio‐recorded and transcribed verbatim by a third‐party company, subject to a confidentiality agreement. Member checking^25^ gave participants an opportunity to amend or add to their transcript; some also provided additional information such as a chronology or narrative of patient experience.

#### 2.1.3 Analysis

The first stage of analysis involved thematic analysis of transcripts and additional information line by line with codes developed deductively (from literature and iterative discussion of initial themes) and inductively.^26^ Seventeen interviews were double‐coded by B. P. W. and R. J. (codes discussed and refined periodically), with B. P. W. coding the remainder. Iterative analysis occurred throughout data collection. B. P. W. and R. J. debriefed after each interview, and throughout data collection and analysis. Analysis was aided by NVivo (release 1.6.1 QSR International).

The second stage involved a focused thematic analysis of coded data about ‘point of access’ to VAD (i.e., how a patient was able to establish a connection facilitating a transition from being outside the VAD system to within it). Therefore, data about barriers *after* a person had connected with the VAD system, or about barriers generally, were excluded. For practical purposes, we determined that access was achieved once a person was connected to a doctor willing and able to assess eligibility or a VAD Care Navigator (or similar position).

B. P. W. analysed these point of access data inductively line by line to identify all potential barriers impeding connection with the VAD system.^26^ Data were thematically grouped into types of barriers and their contributing factors. Analysis included how often particular barriers were raised and their impact on access. Full transcripts were reviewed as appropriate to understand patient chronologies, including when access to the VAD system was achieved. R. J. also reviewed all point‐of‐access data, and preliminary findings were discussed and tested by all authors. Analysis was informed by the team's view that access to VAD for eligible patients who wanted this choice should be available equitably.

Ethics approval was obtained from the Queensland University of Technology Human Research Ethics Committee (2000000270). This research was conducted in accordance with its requirements.

### 2.2 Voluntary Assisted Dying Review Board reports

The Board is the oversight body for the Victorian VAD system.^19^ It must monitor and report on how the Victorian law is operating. All Board reports available on 12 April 2023 were collected via its website and reviewed to identify point of access data. Relevant data were extracted verbatim into a Microsoft Word document and subject to document analysis including content analysis (determining data that are relevant and their meaning) and thematic analysis to identify point of access barriers and contributing factors.^27^

## 3 RESULTS

### 3.1 Sample

#### 3.1.1 Semi‐structured interviews

Twenty‐eight interviews were conducted with 33 participants (32 family caregivers; one patient) (Table 1). Interviews related to the experience of 28 patients (Table 2). Eight participants were recruited during purposive sampling. Recruiting eligible patients was challenging and because only one participant was a patient, the study reports primarily on family caregivers' perceptions. All caregiver interviews were conducted after the patient's death. All eligible participants who contacted the research team were interviewed except for a second prospective patient participant who died before interview. The median interview length was 90 min, with a range of 56–130 min.

**Table 1:** Characteristics of interview participants (*n* = 33).

Characteristics	Number
Age (years), mean (SD)	56.6 (15.1)
20–29	1
30–39	4
40–49	7
50–59	3
60–69	13
70–79	4
80–89	1
Gender	
Female	26
Male	7
Relationship to patient^a^	
Child (including stepchild, child in‐law)	17
Spouse (including de facto partner)	10
Parent	3
Sibling	2
Close friend	1
Self	1

^a^ One participant spoke about two patients so is included in two categories. To further clarify the relationship between interviewees and patients, five of the interviews each involved two family caregivers being interviewed together about their family member's experience of voluntary assisted dying (e.g., a son and daughter‐in‐law speaking about their parent's experience). Two interviews were conducted with different family members separately about the same patient experience.

**Table 2:** Characteristics of patients whose VAD experience was the subject of interviews (*n* = 28).

Characteristic	Number
Age (years), mean (SD)	70.8 (15.4)
20–29	1
30–39	1
40–49	0
50–59	3
60–69	7
70–79	8
80–89	6
90–99	2
Gender	
Female	13
Male	15
Location	
Metropolitan	16
Regional^a^	12
Highest level of education	
Some high school	7
High school	9
University—Diploma	1
University—Undergraduate	7
University—Postgraduate (including graduate diploma)	4
Primary disease, illness, or medical condition	
Cancer	18
Neurological	9
Other	1
Eligibility for VAD and death	
Assessed as eligible	24
Patient died via self‐administered medication	19
Patient died via practitioner administered medication	3
Patient died but did not take medication (natural death)	1
Patient waiting to take medication	1
Patient died before eligibility assessment completed	3
Patient assessed as ineligible and died	1
Timing of VAD (or engagement with process)	
July–December 2019	4
January–June 2020	6
July–December 2020	3
January–June 2021	10
July–November 2021	5

Abbreviation: VAD, voluntary assisted dying.

^a^ One patient classified as regional moved to a metropolitan area during the VAD process.

#### 3.1.2 Voluntary Assisted Dying Review Board reports

The Board produced six reports about 3 years of the VAD system's operation (Table 3). The first report on the first 11 days of the law contains no data and so was not analysed further.

**Table 3:** ‘Reports of operations’ by Voluntary Assisted Dying Review Board (Victoria).

Report number	Data reporting period	Context
1	19 June 2019–30 June 2019	Report was due in first 11 days of the law coming into force, hence no data is reported including in relation to point of access barriers to VAD. Focus is on describing work undertaken by Board to prepare for law commencing. Excluded from sample.
2	19 June 2019–31 December 2019	First report with data (6 Monthly report as required by law).
3	1 January 2020–30 June 2020	6 Monthly report as required by law.
4	1 July 2020–31 December 2020	6 Monthly report as required by law.
5	1 January 2021–30 June 2021	6 Monthly report as required by law.
6	1 July 2021–30 June 2022	First report on annual basis.

Abbreviation: VAD, voluntary assisted dying.

### 3.2 Finding a point of access to the VAD system is challenging

Although nearly all patients whose experience was reported in this study were able to access the VAD system, many participants reported finding a point of access being ‘extremely difficult’ or ‘really hard’. When asked about the biggest challenge in their experience, some participants nominated this issue:

… the biggest challenge about the whole process for us was finding that initial information, knowing what to actually look for and having the right words to be put in a Google search and find it. (Family caregiver 1; patient with cancer)

Some participants observed their background (e.g., highly educated, health or legal training, or being very well‐informed) meant they were more capable than others to find a point of access to VAD. Others considered their access to VAD was due to luck, for example, their usual doctor was already trained and willing. Specific access concerns were expressed for those for whom English is not their first language or older people who may not be Internet literate:

… for someone as educated as her it was … a bit difficult to navigate. So we reflected on how difficult it must be for people who don't have PhDs and don't have first language as English. (Family caregiver 2; patient with cancer)

The Board reports do not specifically identify the concept of a ‘point of access’ to the VAD system being problematic, but their discussion of specific barriers reflects this finding.

### 3.3 Specific point of access barriers

In addition to the global finding that locating a point of access to the VAD system was challenging, participants also identified specific point of access barriers.

#### 3.3.1 Not knowing VAD exists as a legal option

Reflecting inclusion criteria, participants generally reported they or their patient family member knew VAD was legal. This was usually through: the news; longstanding interest in or support for VAD, often linked to VAD group membership; the Internet; family health practitioner expertise; or knowing someone who had sought VAD.

But some participants felt many in the community did not know VAD was an option; older people (who may not be Internet literate) and those from culturally and linguistically diverse (‘CALD’) backgrounds were particularly mentioned. One participant reported discussions at their family member's funeral (who had chosen VAD) showed people did not know it was an option. Participants also described health practitioners not knowing VAD was legal, including one patient's own doctor:

She'd never really heard about it … she wasn't even aware that VAD was a possibility. We actually had to explain to her what she had to do. (Family caregiver 3; patient with neurodegenerative condition)

Participants repeatedly identified the law prohibiting doctors from telling patients about VAD as problematic: if patients do not know and doctors cannot tell them, how can they know about VAD? One participant said:

I think that's okay for people who are educated and know what their options are, but what if you don't know that that's an option for you? (Family caregiver 4; patient with neurodegenerative condition)

Proposed responses to this problem included a ‘broadscale community education program’ and enhanced print or Internet resources, available in hospitals, given by health practitioners when appropriate, or included in disease information packs. Some participants had even taken action to increase community awareness though sharing their story in public forums such as via podcasts, documentaries, newspaper articles, presentations and social media. Participants were motivated to increase awareness about VAD, make ‘getting information a little bit easier’, or ‘demystify it’.

Interview participants recognised that VAD being new meant that wider community awareness had not yet developed. Board reports recognise this too with the fifth report stating that even after 2 years, ‘community awareness and understanding of VAD is still growing’ (p. 16). It records the views of a ‘contact person’ (usually a family member appointed by the patient):

We think that the information needs to be more public so people can have it as choice. It needs to be publicised more. I know it's a grey area, but it's not right for all those people who don't know about it. (Fifth Board Report, p. 16)

#### 3.3.2 Not recognising a person is potentially eligible for VAD

A few participants identified the barrier of not recognising a person's potential eligibility. One issue was an assumption VAD was just for cancer, so other illnesses would not qualify:

Because in our mind we believed it [VAD] was [for] cancer. Cancer, cancer, cancer… My dad didn't have cancer…. He was a man with heart failure… I said ‘I don't think you're eligible’. (Family caregiver 5; patient with heart failure)

Another participant commented that motor‐neurone disease being specifically listed in VAD resources as a potentially eligible condition helped them understand VAD was an option.

A second issue was a lack of awareness that a patient was likely to die within 6 months (or 12 months for neurodegenerative conditions) as per the law's eligibility criteria:

…in terms of the process, well, he never got a prognosis. Because no one actually gets a prognosis, they might be less inclined to possibly apply because they think that unless you have less than six months to live you can't apply. (Family caregiver 6; patient with cancer)

Another participant said ‘no‐one had said you're going to die within six months’ and felt this contributed to delay in starting the process to find a point of access to VAD and this patient missing out.

#### 3.3.3 Not knowing the next steps or not being able to achieve them in practice

Even though participants knew VAD existed and usually knew the patient might be eligible, they reported struggling to know the next steps to seek VAD, and to then implement those steps.

In terms of not knowing the next steps, many described doing extensive research, generally on the Internet, before finding an access point to the VAD system:

… we couldn't work out how to find the actual specific information, who was the person that you needed to talk to, to get the specifics of what you had to do or how you had to go about it … it's hard to find the information. … it probably took two to three months of research to get this document [government VAD brochure] … (Family caregiver 7; patient with cancer)

The Fifth Board Report also notes this issue with a contact person saying:

[The applicant] did find some information [about VAD] on the internet, but it was not clear who to speak to initially. The applicant's GP was not sure of the process or contacts either. (p. 8)

Interview participants suggested improving the web presence for VAD to clarify who to contact and how.

Others described that even knowing the next steps to take, they struggled to implement them. For example, some participants described repeated unsuccessful attempts to raise VAD with various health practitioners.

Participants described two main contact points which ultimately facilitated this point of access to the VAD system: a trained doctor willing to assess eligibility and/or a Care Navigator (or local health service coordinator).

Many participants reported challenges locating a *willing doctor*. (We only report here data about the first ‘co‐ordinating’ doctor in the process, and not the second ‘consulting’ doctor as by then there is already system access). This was particularly difficult in the system's early days, when even contact with a Care Navigator may not have been useful because they also were struggling to identify willing doctors. It was especially hard in regional areas where there are fewer doctors.

Most participants reported beginning their VAD journey with a request to their general or family practitioner. This was problematic when that doctor was a conscientious objector, not interested in being involved even if they did not object, unable to participate due to insufficient experience or qualifications, or had not done the VAD training and did not want to. When their doctor was unwilling to assist, participants felt unclear about next steps, particularly if the doctor would not refer to another doctor or provide information about VAD. One participant stated:

The two doctors, local doctors that were assigned to the aged care facility, each of them were conscientious objectors for VAD… Mum was left pretty much searching for herself for a doctor, in a world where she couldn't really leave to go to appointments. (Family caregiver 8; patient with neurodegenerative condition)

Similarly, other participants described their approach:

So my very first move … was to approach the GP. His answer was ‘No. I won't have any part of it.’… A blanket no…. And when I just asked him would he assist and he just said ‘No. I won't’. Then there was a silence. (Patient participant 1; cancer)

Some resorted to personal or professional networks to identify a willing doctor but acknowledged these links were not available to others. Participants suggested a public list of willing doctors be created, but privacy and other implications were identified.

Board reports also repeatedly identified insufficient numbers of trained and willing doctors as an access barrier, including specifically in relation to points of access. For example, in its third report (first half of 2020), the Board shared a contact person comment: ‘Being a new process, it was hard to find information or know how to find a doctor willing to do voluntary assisted dying’ (p. 7). This issue has reportedly improved over time but issues remain, particularly in regional Victoria.

Some Board reports also identified the impact of conscientious objections by health practitioners on point of access. In response, its most recent report ‘strongly supports the consideration that those who do not support voluntary assisted dying be required to make information available that enables potential applicants to contact the Statewide Care Navigator Service…’ (Sixth Board Report, p. 26)

In terms of the other access point, the *Care Navigators*, a few participants described not knowing this service existed or how to contact it:

I wasn't aware of the care navigator. So maybe a bit more publicity, which could be on a little piece of paper which could be provided to the VAD patient in the first instance, saying ‘You can go to this care navigator’, and avoid running around from person to person. (Family caregiver 9; patient with cancer)

The Fifth Board Report (2 years after the law commenced) also notes that not all people applying for VAD or medical practitioners were aware of the Care Navigators.

A final barrier to operationalising access was *institutional objection*. Although regularly raised by interview participants,^7^ this was generally not in relation to point of access but rather after connection with the VAD system. The limited point of access data about institutional objections tended to be about unwillingness to discuss VAD. Further, while falling short of a formal objection to VAD, there were some reports of institutional stigma or reluctance to engage:

… a couple of times dad brought it up, once with the registrar or whoever he was seeing that day, and once or twice with the social workers or the coordinators. … it was a bit of a ‘We don't do that here. We don't really talk about that here’, and that's when they gave me the VAD navigator's number. … like they weren't allowed to talk about it there. (Family caregiver 10; patient with neurodegenerative condition)

#### 3.3.4 Challenges with patients being required to raise the topic of VAD first

Participants reported difficulties requesting VAD from their doctor. While linked with barriers above, this issue was so prominent in the data it warrants separate consideration. There were four connected components to this barrier.

The first consolidates the above discussion about earlier barriers: the challenge of knowing the law requires patients to raise VAD as doctors cannot raise it first. Participants identified that unless patients know the law requires them to ask about VAD, they may not do this. Indeed, patients may be waiting for their doctors to raise VAD, which they are legally prohibited from doing.

The Board also recognised this: ‘medical practitioners, applicants [patients] and contact people [generally family members] have constantly highlighted this as a barrier to accessing voluntary assisted dying in Victoria.’ (Sixth Board Report, p. 30)

The second component was the practical difficulty patients experienced broaching VAD with their doctor, even when aware it was a legal option and that they had to ask for it first:

I remember him being nervous … it was a big thing for him to bring that up with his doctor. Like that's not an easy conversation to have. And he had a really good relationship with his doctor who was … easy to chat with, but … he still found that stressful. (Family caregiver 11; patient with cancer)

Some participants witnessed patients experience particular difficulty making this request when seeing a new doctor for the purpose of VAD (e.g., because their usual doctor would not provide VAD).

A third component was the need for patients to use sufficiently clear words so the doctor could conclude VAD had been raised. Several participants described failed attempts to ‘use the words’:

I guess he didn't know what he had to ask and how he needed to ask it… he wasn't using the right words, so they couldn't then say ‘Are you talking about assisted dying?’ He clearly said: ‘Can't someone just give me a needle and be done with it?’ Even that was almost just joked off by the registrars … we went from registrar to registrar to registrar, and we were still not getting the answers that we wanted … Dad then was able to say ‘Mate, if I treated my dog like this, I'd be put in jail. I'm done, how do I end this?’ It wasn't until that that point that [a doctor] actually said: ‘Right, I think I'm catching what you're throwing, how about I put you in touch with the VAD coordinator at the hospital?’ (Family caregiver 1; patient with cancer)

The Board has also noted ‘cases where the applicant has not known what to ask for and has had numerous attempts at the request’ (Sixth Board Report, p. 26).

The final component participants identified was this legal prohibition may contribute to broader stigmatisation of VAD. This specific legal categorisation of VAD as a choice which cannot be openly raised reinforces that VAD is suspect and different from other healthcare choices. This can reduce community awareness and acceptance, and create point‐of‐access barriers:

I think an oncologist whose workload day in, day out is to service people who are dying, I think they have every right, if they feel comfortable after having built rapport with their patient, to bring a suggestion like that [VAD] to the table. It would be an important way to actually sort of normalise VAD almost, as like a treatment option just like pain relief or palliative care. And sure it involves actually assisting someone dying which is the most taboo thing, but the reason that it's taboo is because it's treated differently. (Family caregiver 12; patient with cancer)

## 4 DISCUSSION

Interview participants and Board reports identify multiple point of access barriers to the Victorian VAD system. Key findings were access for a patient depends on cumulatively:

1. knowing VAD exists as a legal option;
2. knowing they might be eligible for it;
3. being able to identify and successfully take the next steps to connect with the VAD system; and
4. successfully raising VAD with a doctor.

Box 2 draws together these barriers and contributing factors underpinning them, and proposes some responses to address them, including drawing on possible solutions from the data. We also discuss three key responses below.

BOX 2 **Barriers to accessing the VAD system, contributing factors and example responses^a^**

**Table:** 

Barrier	Contributing factors	Example responses
Not knowing VAD exists as a legal option	Registered health practitioners legally prohibited from raising VAD as an option. Limited health literacy, especially for some diverse populations such as those from CALD backgrounds. Limited Internet proficiency, especially for older people.	Repeal legal prohibition on raising VAD. Community awareness initiatives about VAD as a legal option (including specific strategies to promote community awareness in CALD populations developed in consultation with organisations that support them). Enhanced Internet resources both in terms of their clarity and being able to find the resources on the web (including search engine optimisation). Enhanced print resources, and more widely available e.g. in hospitals and general practice clinics.
Not recognising a person is potentially eligible for VAD	Registered health practitioners legally prohibited from raising VAD as an option. Not knowing that VAD is available for a range of qualifying illnesses, other than just cancer. Not knowing a person's prognosis (eligibility criteria requires anticipated death within 6 months or 12 months for neurodegenerative conditions).	Repeal legal prohibition on raising VAD. Community awareness initiatives about VAD, which include information about who may access VAD. Resources should not only include cancer but other diseases as well and disease‐specific organisations should be engaged in these initiatives. When a patient may be eligible for VAD, the treating doctor should give consideration to sensitively discussing likely prognosis and treatment options so a patient can be aware that they may be eligible for VAD. (This discussion is currently made more difficult because of the legal prohibition on raising VAD but should include sensitively raising the option of VAD if appropriate and the legal prohibition is repealed).
Not knowing the next steps or not being able to achieve them in practice	Not being able to find information about the next steps needed to connect with the VAD system.	Enhanced Internet resources both in terms of their clarity and being able to find the resources on the web (including search engine optimisation). Also enhanced print resources. These resources should include clear guidance on steps needed and easily locatable relevant contact details to progress a request for VAD.
	Not being able to identify a trained and willing doctor to assess VAD eligibility.	Require nonparticipating doctors to refer patients to a willing doctor, or alternatively provide contact details of the VAD Care Navigators to ensure connection with the VAD system. Increase the pool of trained and willing doctors, particularly in general practice given it is likely to be the first point of contact. This could be done in a range of ways including: making the criteria for doctors to participate in VAD less onerous (to our knowledge, Victoria has the most onerous requirements internationally); and providing adequate time and/or remuneration for doctors to be involved in VAD and for them to undertake the mandatory training.
	Not knowing about the VAD Care Navigators (or local health service coordinator) or being able to find them.	Enhanced Internet resources both in terms of their clarity and being able to find the resources on the web (including search engine optimisation). Also enhanced print resources. These resources should include clear guidance on steps needed and easily locatable relevant contact details for VAD Care Navigators. Require nonparticipating doctors to refer patients to a willing doctor, or alternatively provide contact details of the VAD Care Navigators to ensure connection with the VAD system.
	Institutional objection.	Require objecting healthcare institutions to at least provide information about VAD if patients ask about it, including the contact details of the VAD Care Navigators (other steps may also be required to address other access issues but provision of information should be sufficient to address point of access issues).
Challenges with patients being required to raise the topic of VAD first	Not knowing that registered health practitioners are legally prohibited from raising VAD as an option.	Repeal legal prohibition on raising VAD. Community awareness initiatives about VAD, which include information that it must be the patient who raises the issue of VAD.
	Raising VAD with a doctor is difficult.	Repeal legal prohibition on raising VAD. Conversation guides to support patients wishing to raise VAD, easily locatable on the web. Further training for doctors about VAD, so they can recognise when a patient may be requesting VAD, and if the legal prohibition is repealed, so they are comfortable raising VAD if appropriate. There should also be training about end‐of‐life conversations generally to make such discussions easier for patients.
	Not knowing the words sufficient to have raised the topic of VAD so it may be discussed with a doctor.	Repeal legal prohibition on raising VAD. Conversation guides to support patients wishing to raise VAD, easily locatable on the web. Further training for doctors about VAD so they can recognise when a patient may be requesting VAD, and if the legal prohibition is repealed, so they are comfortable raising VAD if appropriate.
	Stigmatisation of VAD because, unlike other healthcare, it cannot be openly raised with patients.	Repeal legal prohibition on raising VAD. Community awareness initiatives about VAD, which include addressing potential stigmatisation of VAD.

Abbreviations: CALD, culturally and linguistically diverse; VAD, voluntary assisted dying.

^a^ These responses are proposed with acknowledgment that in Victoria (the site of this research), there is already some existing work undertaken to address the issues identified. See for example the consumer‐facing website: Department of Health, Victoria State Government. Community and consumer information. Accessed July 14, 2023. https://www.health.vic.gov.au/patient-care/community-and-consumer-information

But first, we observe that this lack of patient and community awareness about VAD is a health literacy issue. We are not aware of research specifically examining health literacy about VAD (some studies make cursory mentions), but the problems identified by participants reflect this wider literature. For example, low health literacy leads to difficulties accessing healthcare, including difficulty finding healthcare providers or establishing a usual source of care.^17^, ^28^ Low health literacy also causes delays seeking care because individuals may not recognise symptom development (in our study, prognosis) that should motivate action.^28^

A case can be made to improve health literacy about VAD (like other healthcare), but this is particularly compelling when potentially eligible patients are legally prevented from being informed about VAD by their doctor, as in Victoria.

### 4.1 Repeal legal prohibition on raising VAD

The legal prohibition on registered health practitioners raising VAD with patients was the most significant challenge for point of access. These concerns reflect research on participating doctors' perspectives on the Victorian VAD system.^9^, ^10^, ^11^

Considering these findings and other critiques of this legal prohibition,^5^, ^8^, ^11^, ^20^ we propose it be repealed. This aspect of Victorian law is inconsistent with general legal duties to inform patients of their choices and was unique internationally when legislated, although has since been copied by South Australia and New Zealand. Its intention was to avoid coercion or undue influence,^29^ but these concerns are addressed in other safeguards, including two independent medical assessments of a person's choice being voluntary. Given its adverse impact on access, we consider the prohibition is unjustifiable and health practitioners should be able to inform their patients of all possible choices, which may include VAD. This is particularly important because some patients may be waiting for their health practitioner, as with end‐of‐life discussions generally,^30^ to raise VAD as an option. More open discussions about VAD may also help normalise it as an end‐of‐life choice and reduce stigma. This may, over time, improve community awareness of VAD and consequently patient access.

However, law reform alone to permit raising VAD will be insufficient. Reflecting medical discomfort with discussing death and dying generally,^31^ some doctors, even if legally able, may not feel comfortable raising VAD, or they may create an environment where patients find asking about VAD difficult.^32^, ^33^ We propose, therefore, training about VAD and how to discuss it should be included in medical school curricula and continuing professional development training. In jurisdictions with a legal prohibition on raising VAD, that training should include guidance on how to recognise when a patient has initiated a VAD discussion.

### 4.2 Require nonparticipating doctors to refer or provide information to patients, and require institutions to provide information to patients

Participants whose doctors did not want to participate in VAD (including because of conscientious objection) described uncertainty about next steps. We propose that VAD systems should require doctors to connect patients to the system either via a referral to a doctor willing to assess eligibility or the contact details of Care Navigators (if such a role exists). This duty already exists in other Australian states' VAD laws^3^ and we propose the Victorian law be amended accordingly.

Drawing on the Canadian experience, education and monitoring may be needed to ensure this duty is known and followed. To illustrate, despite medical college policy in provinces such as Ontario, British Columbia and Nova Scotia requiring doctors who are unwilling to provide VAD based on their beliefs to provide an effective referral, information, or transfer of care^34^, ^35^, ^36^ (the Ontario policy being legally upheld as valid in court^37^), access problems remain. For example, Nova Scotia research has shown a key delay in starting the VAD process is a failure or refusal of unwilling providers to make a referral.^14^

Finally, while imposing referral or information duties on doctors may address some access issues, some VAD requests may be directed to other health practitioners (such as nurses) who have a conscientious objection. Further, some institutions object to participating in VAD.^7^ To ensure requests for information are met, a wider approach is warranted, and we consider this is best addressed at an institutional level. Therefore, we propose a legal obligation on healthcare institutions to, at the very least, provide information about VAD (including Care Navigators' contact details) if requested by a patient or resident.^38^

### 4.3 Community awareness initiatives to enhance health literacy about VAD

Interview participants repeatedly called for community awareness initiatives to address perceived deficits in public knowledge about VAD. Indeed, many sought to tackle this themselves, for example, by publicly sharing their VAD experience.^18^

Reflecting our findings, we consider such community awareness initiatives should: explain VAD is a legal end‐of‐life choice; outline eligibility criteria and potentially qualifying illnesses; explain VAD must be raised by the patient first, and that clear words must be used (if required by local law); and address stigma that may be attached to choosing VAD which could impede access. We recognise such initiatives must be sensitively designed to ensure the focus is on information provision and that community members do not feel induced or directed to access VAD.

Such initiatives must also recognise specific needs of diverse populations, reflecting the wider health literacy focus of ensuring equality of access to care.^39^ Our participants raised concerns about awareness of VAD by older persons, people from CALD backgrounds, and those with lower levels of education; such concerns are also reflected in broader health literacy literature.^17^, ^28^, ^40^ It is perhaps not surprising that, internationally, research has found that people accessing VAD are more often from higher socioeconomic backgrounds,^41^, ^42^, ^43^, ^44^, ^45^ and have higher education levels.^43^, ^44^, ^45^, ^46^, ^47^ This also reflects data from the Sixth Board Report that VAD applicants were ‘considerably more highly educated’ than the general Victorian population (p. 14). Information should be tailored to the needs of specific populations so they can make the same choices about VAD. For example, older persons with limited Internet literacy may prefer physically accessible print resources in locations they are likely to seek healthcare while other groups may need resources translated into different languages and presented in a culturally safe way.

These community awareness initiatives could be connected with existing work to promote end‐of‐life care discussions, advance care planning and wider ‘death literacy’.^48^ Embedding VAD within a suite of wider end‐of‐life choices will help reduce potential stigma.

Finally, while community awareness initiatives are most likely government‐led, they should proactively harness other organisations who can amplify and share this information. Mainstream media is an obvious example but others include disease‐specific organisations (particularly those where VAD eligibility may be less obvious), patient advocacy groups, older persons' associations, and organisations for CALD people. Other organisations include places where healthcare is accessed such as general practice clinics, hospitals, and residential aged care facilities. Also, there is an argument that some of these nongovernment organisations should lead such initiatives because their position outside the machinery of government avoids concerns about inducement from the state to consider VAD.

### 4.4 Limitations

While able to highlight point of access barriers, our data is from individuals who connected with the VAD system. More research is needed with those who were not able to connect, although this is a challenging cohort to recruit and interview. Another limitation is that interviews were primarily with family caregivers. Although proxies are a reliable source regarding quality of end‐of‐life services, demonstrating high concordance with patient views,^49^ their perceptions may differ from patients' due to grief, bereavement, and their relationship with the patient they were supporting.^49^, ^50^, ^51^ More research directly with patients seeking VAD is needed. Our sample may also be more favourably disposed towards VAD, given some recruitment was via patient interest groups. Further, as a qualitative study, this research does not make claims about the frequency of point of access issues but instead reports on the breadth of experiences described.

In addition, this research occurred in Victoria, Australia which has a specific law and practice framework. Further, this study was conducted in the system's early days (e.g., just under 3 years at the time of interviews and some interviewees reported earlier experience; likewise Board reports reflect experience at the date of publication). Access issues are likely to improve over time as the system becomes more established (see below).

### 4.5 Concluding observations: Wider implications for VAD systems

VAD systems aim to provide choice for eligible patients seeking assistance to die, while also ensuring safety, for example, by excluding people who do not meet eligibility criteria.^5^ However, sensitivity about VAD during legislative debates may lead to a primary focus on safety without due regard to the need for access.

This occurred in Victoria where sufficient votes in Parliament depended on the law's narrow eligibility criteria, assessed through a very rigorous process with numerous safeguards.^52^ Less consideration was given to access issues,^5^, ^53^ contributing to the problems identified above. This research demonstrates the importance for other jurisdictions contemplating reform to include access (not just safety) as a core consideration when designing and implementing VAD laws.

From a practical perspective, access issues may be greater in a VAD system's early days when there is less awareness of VAD and fewer doctors to assess eligibility. This reflects challenges in Canada,^13^, ^14^, ^15^, ^16^ and in Oregon where Chin et al.^54^ found that 40% of patients seeking VAD in the first year of assisted dying were unable to initiate the process with the first provider approached. Over time, these issues may settle with greater community awareness of VAD but also better integration within wider end‐of‐life care.^55^ When legalising VAD or during the implementation phase, jurisdictions should proactively integrate VAD into the wider healthcare system to support access.

These findings also suggest a particular focus on supporting access to VAD through primary care, namely general or family practitioners. These practitioners were usually the first contact point for information or access about VAD, reflecting the international experience.^56^, ^57^ Specific efforts to support VAD information and provision in the primary care sector are therefore important to enhance access, including publicly funded remuneration^58^ and targeted continuing professional development opportunities for general practitioners.

Finally, there is very little empirical research investigating this initial point of access to VAD systems, and that which exists usually considers it as a subset of broader findings.^13^, ^14^, ^15^, ^16^ Patients who did not connect with the VAD system, but may have wished to (including if they knew it existed), are difficult to identify and conduct research with. Indeed, this study's cohort *was* able to connect with the VAD system. Existing data collection by oversight bodies will also generally not capture the experiences of people who fail to achieve access. This is an established methodological challenge across research examining access to healthcare, described by Levy and Janke^17^ as: ‘focusing on patients who are already in the door [which] misses a piece of the problem’. More work is needed to understand and address point of access barriers to VAD, including for those who do not ‘make it in the door’. This may point to the need for a community‐wide survey (or of particular populations likely to seek VAD such as cancer patients) to determine baseline levels of knowledge about VAD and potential barriers to seeking relevant information.

Optimal VAD systems are not only safe but also accessible for eligible patients. This access depends on an ability to successfully connect to the VAD system. Yet our research demonstrated this was challenging, even for a cohort who were ultimately successful in commencing the VAD process. We call for law changes to address barriers to discussing and knowing about VAD, health system reform to enhance access via primary care, and community awareness initiatives to build health literacy about VAD.

## AUTHOR CONTRIBUTIONS

Data were collected and analysed by Ben P. White and Ruthie Jeanneret. Lindy Willmott had access to all data. Ben P. White wrote the first draft of the manuscript. Ben P. White, Ruthie Jeanneret and Lindy Willmott made substantive revisions to iterative drafts of the manuscript. Ben P. White finalised the text, which was approved by all authors.

## CONFLICT OF INTEREST STATEMENT

Ben P. White and Lindy Willmott were engaged by the Victorian, Western Australian and Queensland governments to provide the legislatively mandated training for health practitioners involved in voluntary assisted dying. Ruthie Jeanneret was employed on the Victorian, Western Australian and Queensland training projects. Ben P. White is a session a member of the Queensland Civil and Administrative Tribunal, the quasi‐judicial review body which has jurisdiction over some voluntary assisted dying matters. Lindy Willmott is a member of the relevant oversight body in Queensland, the Voluntary Assisted Dying Review Board. All views expressed in this article are those of the authors and not the organisations they are affiliated with.

## ETHICS STATEMENT

Ethics approval was obtained from the Queensland University of Technology Human Research Ethics Committee (2000000270). This research was conducted in accordance with the Australian National Health and Medical Research Council's National Statement on Ethical Conduct in Human Research 2007 (updated 2018). This research was conducted in accordance with the requirements of this ethics approval. All persons interviewed gave free and informed consent to participation in this research.

## Supporting information

Supporting information.

## Data Availability

The data that support the findings of this study are available on request from the corresponding author. The data are not publicly available due to privacy or ethical restrictions. The interview guide is available in Supporting Information File.
